# Genome-Wide Identification and Expression Analysis of 1-Aminocyclopropane-1-Carboxylate Synthase (*ACS*) Gene Family in *Chenopodium quinoa*

**DOI:** 10.3390/plants12234021

**Published:** 2023-11-29

**Authors:** Lu Yin, Xia Zhang, Aihong Gao, Meng Cao, Dongdong Yang, Kexin An, Shanli Guo, Haibo Yin

**Affiliations:** 1 College of Life Sciences, Yantai University, Yantai 264005, China; yinlu0127@s.ytu.edu.cn (L.Y.); zhangxia@ytu.edu.cn (X.Z.); gahgah123@s.ytu.edu.cn (A.G.); caomeng123@s.ytu.edu.cn (M.C.); 19353512768@s.ytu.edu.cn (D.Y.); aankexin@s.ytu.edu.cn (K.A.); 2 College of Grassland Sciences, Qingdao Agricultural University, Qingdao 266109, China; 3 High-Efficiency Agricultural Technology Industry Research Institute of Saline and Alkaline Land of Dongying, Qingdao Agricultural University, Dongying 257300, China; 4 Key Laboratory of National Forestry and Grassland Administration on Grassland Resources and Ecology in the Yellow River Delta, Qingdao Agricultural University, Qingdao 266109, China

**Keywords:** *C. quinoa*, ethylene, *ACS* genes, expression patterns, abiotic stress

## Abstract

Ethylene plays an important role in plant development and stress resistance. The rate-limiting enzyme in ethylene biosynthesis is 1-aminocyclopropane-1-carboxylic acid synthase (ACS). *C. quinoa* (*Chenopodium quinoa*) is an important food crop known for its strong tolerance to abiotic stresses. However, knowledge regarding the *ACS* gene family in *C. quinoa* remains restricted. In this study, we successfully identified 12 *ACS* genes (*CqACSs*) from the *C. quinoa* genome. Through thorough analysis of their sequences and phylogenetic relationships, it was verified that 8 out of these 12 CqACS isozymes exhibited substantial resemblance to ACS isozymes possessing ACS activity. Furthermore, these eight isozymes could be categorized into three distinct groups. The four remaining *CqACS* genes grouped under category IV displayed notable similarities with *AtACS10* and *AtACS12*, known as amido transferases lacking ACS activity. The CqACS proteins bore resemblance to the AtACS proteins and had the characteristic structural features typically observed in plant ACS enzymes. Twelve *CqACS* genes were distributed across 8 out of the 18 chromosomes of *C. quinoa*. The *CqACS* genes were expanded from segment duplication. Many cis-regulatory elements related with various abiotic stresses, phytohormones, and light were found. The expression patterns of *ACS* genes varied across different tissues of *C. quinoa*. Furthermore, the analysis of gene expression patterns under abiotic stress showed that *CqACS* genes can be responsive to various stresses, implying their potential functions in adapting to various abiotic stresses. The findings from this research serve as a foundation for delving deeper into the functional roles of *CqACS* genes.

## 1. Introduction

The growth and development of plants are intricately regulated by phytohormones, including ethylene [1,2,3,4], jasmonic acid [5,6], cytokinin [7,8], gibberellin [9,10], abscisic acid [11,12], and brassinosteroids (BRs) [13,14]. Being a gaseous phytohormone, ethylene plays diverse roles in various physiological processes, encompassing the initiation of root growth, the maturation of fruits [15], the senescence and abscission of flowers and leaves [15,16], as well as responses to biotic and abiotic stresses, including pathogen infection, hypoxia, cold, heat, salinity, and drought stress [17,18]. Understanding the molecular mechanisms underlying ethylene biosynthesis and its regulation plays a pivotal role in deciphering the complex orchestration of plant growth and the management of stress responses.

In the ethylene biosynthesis pathway, commonly known as the Yang cycle, ethylene production involves two key steps: (1) the conversion of S-adenosyl methionine to the ethylene precursor 1-aminocyclopropane-1-carboxylic acid (ACC) through the action of ACC synthase (ACS); and (2) the conversion of ACC to ethylene, carbon dioxide, and cyanide by ACC oxidase [19,20]. Among these steps, ACS catalyzes the rate-limiting reaction, making it a critical enzyme in ethylene biosynthesis [21]. In plants, it is crucial to precisely regulate ethylene biosynthesis through transcriptional and post-translational regulation of ACC synthase and ACC oxidase [22]. In the commonly studied plant *Arabidopsis thaliana*, the *ACS* gene family was extensively investigated. Nine ACS enzymes (AtACS1, AtACS2, AtACS4–9, and AtACS11) possess catalytic activity. *AtACS3* is a pseudogene, while AtACS10 and AtACS12 exhibit aminotransferase activity [23,24,25]. These ACS proteins display distinct structural features and are classified into three types based on their domains and target sites. ACS enzymes in Type I (AtACS1, AtACS2, and AtACS6) exhibit specific regions that serve as phosphorylation sites for calcium-dependent protein kinases (CDPKs) and mitogen-activated protein kinases (MAPKs), implying that their functions are regulated by phosphorylation processes. Type II ACSs (AtACS4, AtACS5, AtACS8, AtACS9, and AtACS11) possess distinct sites in their C-terminal region that are targeted by calcium-dependent protein kinases (CDPKs) and E3 ligases [23,24,25], implicating their involvement in protein–protein interactions and ubiquitination. For Type III ACS (AtACS7), there is no specific target site. However, its degradation is facilitated by interaction with E3 ubiquitin–protein ligase XBAT32 [26,27] or through dephosphorylation of protein phosphatase 2C [28]. The stability and activity of ACS proteins are also regulated by interactions with 14-3-3 proteins, which protect them from degradation [29].

Earlier research established that ACS enzymes are the product of a gene family with multiple members, and these members are subject to distinct forms of regulation in response to various cues, including signals related to plant growth stages, environmental conditions, and hormonal influences [23,30,31]. In climacteric fruits, *ACS* plays a direct role in regulating the ripening process and influencing the preservation of fruits during postharvest storage. As an example, in apples, the production of ethylene throughout the phases of fruit ripening and softening was under the influence of distinct genetic variations of *MdACS1* (*MdACS1-1* and *MdACS1-2*) and *MdACS3a* [32,33,34,35]. Similarly, in tomato, *ACS2* plays a key role in regulating ripening-specific ethylene biosynthesis. The tomato *acs2-1* mutant (ethylene over-producer) showed accelerated fruit ripening, early seed germination, and faster leaf senescence. Conversely, tomato *acs2-2* mutant (ethylene under-producer) had prolonged fruit ripening, delayed seed germination, and slower leaf senescence [36]. In some vegetable crops, *ACS* genes were involved in sex differentiation and flower development, including *CpACS27A* in squash, *CsACS1G*, *CsACS2*, and *CsACS11* in cucumber, and *CmACS7* and *CmACS11* in melon, and *CitACS4* and *ClACS7* genes in watermelon [37,38,39,40]. In maize, the ethylene biosynthetic gene *ZmACS7* plays crucial roles in regulating plant height and leaf angle [41]. Furthermore, recent studies showed that overexpression of *CiACS4* in tobacco and lemon led to dwarf phenotypes by increasing ethylene release and inhibiting gibberellin biosynthesis [42].

Diverse regulatory mechanisms were extensively documented with regards to the roles that aminocyclopropane-1-carboxylic acid synthases (*ACSs*) play in responding to different abiotic stresses. In *Arabidopsis*, *ACS2*, *ACS6*, *ACS7*, and *ACS9* were induced during hypoxia [30]. In tobacco, the expression of *ACS* gene was induced in hypoxic lateral roots, but not in adventitious roots [43]. Mutations in *OsACS1* or *OsACS2* led to a decrease in ethylene biosynthesis and lateral root elongation under Pi deficiency conditions, indicating that *OsACS1* and *OsACS2* were involved in Pi deficiency-induced adaptive responses with a significant role in altering root system architecture [44]. Accumulation of *ACS2* and *ACS6* transcripts in *Arabidopsis* under cadmium stress increases ethylene synthesis [45]. Under moderate drought conditions, the activation of ACS2 and/or ACS6 increased stomatal density and clustering rate on the leaf epidermis of *Arabidopsis* by accumulating ACC and increased the survival risk of seedlings under drought escalation conditions [46]. The *OS-ACS5* gene was induced during short-term and long-term complete submergence of seedlings. *OS-ACS5* mRNA was localized in specific cells and tissues during normal development and complete submergence [47]. Hormone crosstalk plays an important role in enabling plants to respond plastically to specific developmental or environmental inputs [48]. The biosynthesis of ethylene is regulated by other plant hormones at the transcriptional and post-transcriptional levels. The increase in auxin levels triggers transcriptional activation of the *ACS* gene subgroup, leading to an increase in ethylene biosynthesis [49]. Cytokinin, brassinosteroids, gibberellic acid, and strigolactone increased ethylene production in etiolated *Arabidopsis* and rice seedlings by regulating the protein stability of ACS but did not change the transcriptional level of *ACS* [50,51,52].

*C. quinoa*, a member of the Amaranthaceae family, is a traditional local crop, well known for its remarkable tolerance to abiotic stresses, including frost, drought, and salinity [53]. However, the *ACS* genes in *C. quinoa* were not thoroughly investigated, leading to a limited understanding of the *CqACS* gene family in *C. quinoa*. To bridge this knowledge gap, we conducted a genome-wide study aimed at identifying putative *ACS* family genes in *C. quinoa*. We systematically analyzed the evolutionary relationships, gene structure, conserved motifs, chromosome location, collinearity relationships, and cis-regulatory elements of the *CqACS* genes. Moreover, we investigated the expression patterns of *CqACS* genes in different tissues and under various abiotic stress treatments. Our findings provide valuable insights into the diversity of the *CqACS* gene family and its potential roles in ethylene biosynthesis and stress responses in *C. quinoa*. Identifying and characterizing *CqACS* genes lay the foundation for further functional studies in order to understand the specific roles of individual genes in ethylene-mediated processes and stress tolerance mechanisms in this resilient crop.

## 2. Results

### 2.1. Identification and Physicochemical Properties of CqACS Gene Family

To comprehensively identify the potential *ACS* genes in *C. quinoa*, whole-genome scanning was performed to search these genes using the BlastP method and the hidden Markov model (HMM). A total of 12 *ACS* genes were identified in the genome of *C. quinoa*. These genes were named based on homology with *Arabidopsis ACS* genes and detailed information on these *ACS* genes was shown in Table 1. The isoelectric points (PI) of CqACS proteins ranged from 5.27 (CqACS7a) to 7.99 (CqACS9a and CqACS9b). The number of amino acids of CqACS proteins ranged from 359 (CqACS12a) to 548 (CqACS10a). The predicted grand average of hydropathy (GRAVY) values showed that ten of them were less than zero, suggesting they were hydrophilic, while the rest of them were 0.018 (CqACS12a) and 0.012 (CqACS12b), which meant they were hydrophobic. Subcellular localization prediction revealed that four CqACS proteins were positioned in the nucleus, three in the chloroplast, and two in plasma membrane, while one CqACS protein was found in each of the cytosol, vacuolar membrane, and cytoskeleton.

### 2.2. Phylogenetic Analysis of the ACS Gene Family in Quinoa

**Figure 1:**
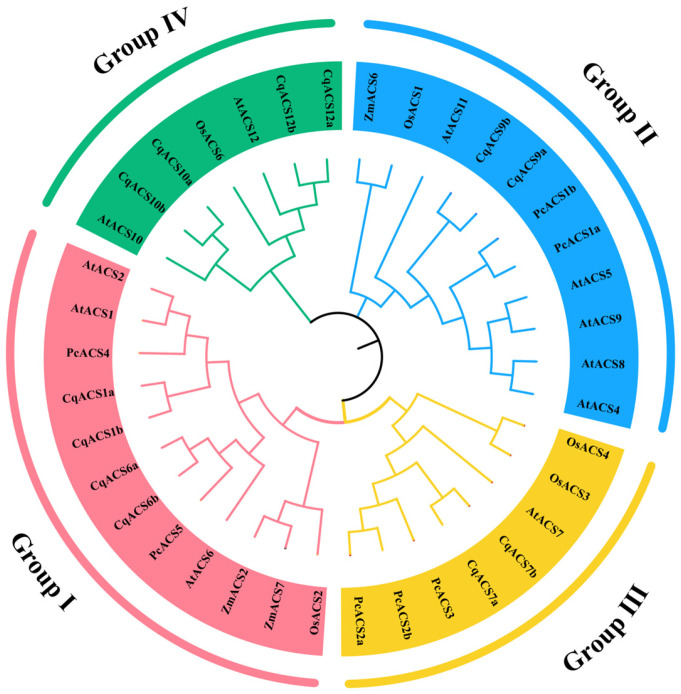
Phylogeny relationship of the ACS proteins in *C. quinoa* and other species. The neighbor-joining phylogenetic tree was constructed based on a multiple sequences alignment of 38 ACS protein sequences from five species including *Zea mays* (*ZmACS*), *Pyrus communis* (*PcACS*), *Chenopodium quinoa* (*CqACS*), *Oryza sativa* (*OsACS*), and *A. thaliana* (*AtACS*), with 1000 bootstraps and model of a Poisson model.

To further understand the *CqACS* gene family pedigree and functional characteristics, the phylogenetic tree was constructed with the ACS proteins of five species, including monocotyledon-representative plants *Zea mays* (three *ZmACSs*), *Oryza sativa* (five *OsACSs*), dicotyledon-representative plants *Arabidopsis thaliana* (eleven *AtACSs*), *Pyrus communis* (seven *PcACSs*), and *C. quinoa* (twelve *CqACSs*). Using MEGA7.0 software, we created a phylogenetic tree that analyzed the evolutionary relationships among 38 full-length protein sequences of ACS genes. As a result, as shown in Figure 1, all 38 ACS proteins were found to cluster into four subgroups. Group I consisted of 12 members, Group II consisted of 11 members, Group III consisted of 8 members, and Group IV consisted of 7 members. Among the CqACSs, CqACS1a, CqACS1b, CqACS6a, and CqACS6b were grouped into Group I, CqACS9a and CqACS9b were in Group II, and CqACS7a and CqACS7b were in Group III. CqACS10a, CqACS10b, CqACS12a, and CqACS12b showed close similarity to AtACS10 and AtACS12, which are included in Group IV and were presumed to function as amino acid transferases without ACS activity [23]. These four subgroups included monocotyledonous and dicotyledonous plants, suggesting that *ACS* genes appeared before the separation of monocotyledonous and dicotyledonous plants.

### 2.3. Gene Structure and Conserved Motif Analysis of CqACSs

**Figure 2:**
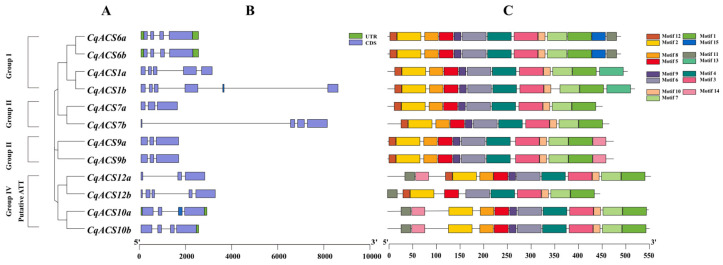
Phylogenetic relationships, exon–intron structure, and conserved protein motifs of *CqACSs*. (**A**) A dendrogram illustrating the evolutionary relationships among *CqACSs* based on their sequences. According to phylogenetic relationships, 12 *CqACSs* were clustered into four groups (I–IV). (**B**) The arrangement of exons and introns in *CqACSs* is depicted, with UTR regions represented by green boxes, exons by violet boxes, and introns by black lines. (**C**) *CqACSs* showcase a diverse motif composition, with each unique motif represented by differently colored boxes.

The exon–intron configurations of *CqACS* genes were studied to understand the structural evolution of the *CqACS* gene family. In general, the genomic sequences of *ACS* may contain 1–5 introns, with the position of each intron conserved [54]. As shown in Figure 2B from our research, the 12 *CqACS* genes demonstrated a variability in exon number, spanning from three to six exons (four with three exons, five with four exons, two with five exons, one with six exons). Among them, CqACS2b has six exons, with the highest number of exons. This indicated that exon loss and acquisition events occurred during the evolution of the *CqACS* gene family, which may lead to functional diversity of the *CqACS* genes. Furthermore, our observations indicated a resemblance in the exon number between *CqACS* genes and the *ACS* family genes of *A. thaliana* [23], as well as those of watermelon and melon [55]. Additionally, we noted a correlation where genes with closer evolutionary relationships exhibited similarities in exon length and distribution. This alignment in exon characteristics could serve as supplementary substantiation for the inferred phylogenetic connections within a specific gene family.

We studied the full-length protein sequences of the 12 CqACSs to identify their conserved motifs (Figure 2C). Fifteen motifs were identified among the ACSs and are listed in Table S1. Among them, motifs 1–10, 12, 14, and 15 are conserved domains associated with aminotransferase domains. Motifs 1–10 were identified across all CqACS proteins, signifying a commonality. CqACS6a and CqACS6b had an exclusive motif 15, while CqACS1a and CqACS1b contained a unique motif 13; CqACS12b lacked motif 14. These motif disparities could potentially underpin the functional divergence among various CqACS proteins. Through a comprehensive multiple sequence alignment, we observed the resemblance of CqACS proteins to AtACS proteins, with adherence to the characteristic structure of plant ACS (Figure S1). Notably, all CqACS isozymes retained the seven conserved boxes that are recurrent in ACS proteins across *Arabidopsis* and other plant species [23,51,56]. The eleven conserved residues that are authentic to ACS enzymes [23] also existed in the members of quinoa *ACS* gene family, but some residues changed. For example, the tyrosine (Y) residue in box 2 was replaced by phenylalanine (F) in CqACS10a, CqACS10d, CqACS12a, and CqACS12b, whereas in AtACS10 and AtACS12, it was replaced by serine (S) and phenylalanine (F), respectively. The tyrosine (Y) residue in box 2 is crucial for anchoring the PLP cofactor onto the ACS apoenzyme. This tyrosine residue also could interact with the active-site lysine (K) residue located in box 5 to form a covalent Schiff base with the attached PLP in unligated enzyme [57]. Interestingly, the active-site lysine (K) residue in box 5 was replaced by glutamate (E) residue in CqACS10a and CqACS10b. Moreover, the conserved glutamate (E) residue responsible for substrate specificity [58] in box 1 was present in all members of the ACSs, except CqACS10a and AtACS10, where it was replaced by lysine (K) and glutamine (Q), respectively. Therefore, similar to AtACS10 and AtACS12, CqACS10a, CqACS10b, CqACS12a, and CqACS12b are expected to catalyze reactions that do not include ethylene production but were still considered ACS enzymes due to their membership in the amino acid aminotransferase family. Consequently, the *C. quinoa ACS* gene family consists of eight authentic ACS (*CqACS1a/1b*, *CqACS6a/6b*, *CqACS7a/7b*, and *CqACS9a/9b*) and four amino acid transferases (*CqACS10a/10b*, and *CqACS12a/12b*).

According to the amino acid sequence of the C-terminal region, AtACS proteins were classified as three types (types 1, 2, and 3) [23]. As shown in Figure S1, type 1 ACS proteins, including CqACS1a and CqACS1b, possessed the serine residue in the “RLSF” motif, which is important for CDPK phosphorylation, followed by a long C-terminal tail with the three conserved serine residues serving as targets of MAPK phosphorylation [59]. Type 2 ACS proteins, including CqACS9a and CqACS9b, contained binding motifs (‘WVF’ and ‘RLSF’) for ethylene-overproducer 1 (ETO1) [60]. ETO1 is a E3 ubiquitin ligase and promotes the degradation of ACS proteins via a proteasome pathway. Type 3 ACS proteins, including CqACS7a and CqACS7b, had short C-termini lacking all the key residues for phosphorylation. Interestingly, we found that CqACS6a and CqACS6b possessed a long C-terminal tail with only key residues for MAPK phosphorylation, without the ‘RLSF’ motif for CDPK phosphorylation.

### 2.4. Chromosomal Distribution and Synteny Analysis of CqACS Genes

**Figure 3:**
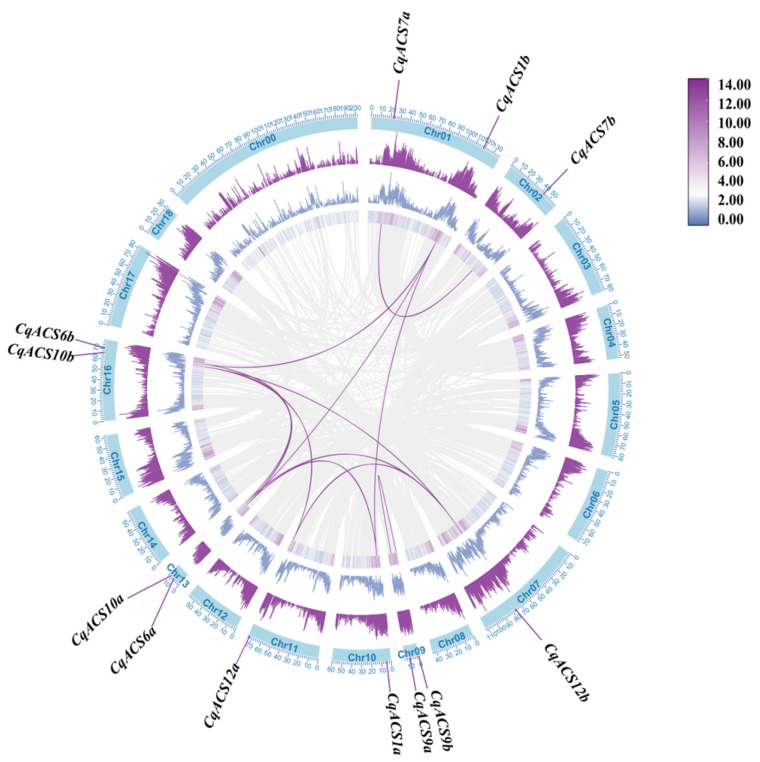
Chromosomal distribution of *CqACS* genes. Moving from the outermost to the innermost circles, the first circle represents chromosome coordinates, while the second, third, and fourth circles illustrate the distribution of gene density; purple lines connect gene pairs.

Referring to the chromosome annotation data of *C. quinoa*, the identified *CqACS* genes were found to be unevenly distributed across eight chromosomes (Table 1 and Figure 3). The analysis revealed that chromosomes 1, 9, 13, and 16 each contained two *ACS* genes, while chromosomes 2, 7, 10, and 11 had one *ACS* gene each. We analyzed the intraspecific collinearity of the *ACS* genes in *C. quinoa*. The gene duplication analysis based on sequence similarity of *CqACS* identified 12 gene pairs and revealed that segmental duplication occurred in 12 gene pairs (Table S2). These findings suggest that segmental duplication may play a major role in driving the expansion of the *ACS* gene family in the *C. quinoa* genome. The Ka/Ks ratios of all gene duplication were also calculated to study the evolutionary selection of *CqACS* gene family. The results show that the values were consistently <1.0 for all gene pairs (Table S2), except for *CqACS1a/CqACS6a* and *CqACS6a/CqACS1b*, indicating that purification selective pressure is the main evolutionary pressure for most *CqACS* genes, which can maintain their functional stability.

**Figure 4:**
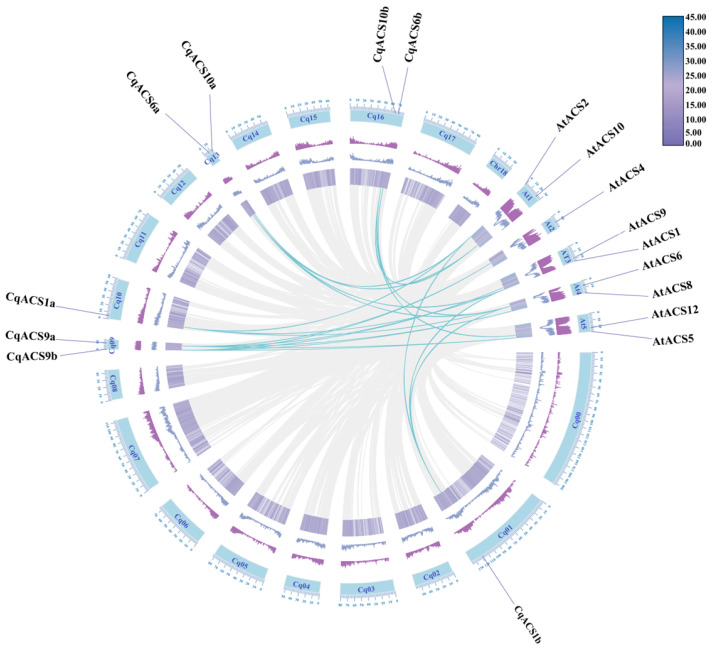
Synteny analysis of *ACS* genes between *C. quinoa* and *A. thaliana*. The collinear blocks generated by the *C. quinoa* and *A. thaliana* genomes are represented by gray lines in the background, while syntenic *ACS* gene pairs are indicated with cyan blue lines.

To further investigate the phylogenetic mechanism of the *CqACS* gene family, we established the whole-genome collinearity relationship between *C. quinoa* and *A. thaliana*. The analysis revealed 22 pairs of orthologous *CqACS* genes between *C. quinoa* and *A. thaliana* (Figure 4, Table S3). Among the 12 *CqACS* genes, 8 *CqACS* genes (8/12) exhibited collinearity with *A. thaliana* (Figure 4). Notably, *CqACS1a*, *CqACS1b*, *CqACS6a*, *CqACS9a*, and *CqACS9b* displayed more than three syntenic gene pairs (Table S3), suggesting their potential significant roles in the evolution of the *CqACS* gene family.

### 2.5. Cis-Elements in the Promoters of CqACSs

Analyzing the cis-elements in promoters helps to understand the precise regulation of genes [61]. In this study, we examined the 2000 bp sequence located upstream of the start codon for each *CqACS* gene, using PlantCARE tools to identify cis-elements. These cis-elements included general transcriptional regulatory elements and functional elements, which play critical roles in the regulation of gene expression.

**Figure 5:**
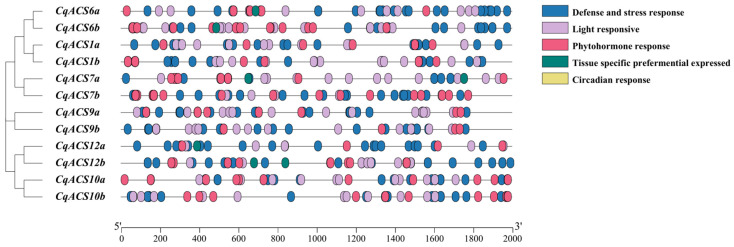
Cis-elements in the promoters of *CqACSs*. Cis-elements possessing comparable functions are represented within identical blocks and color codes.

As shown in Figure 5 and detailed in Table S4, the promoters of *CqACS* genes contained a variety of cis-elements that respond to light, stress, and phytohormones. The stress response elements included those involved in water stress response (AT-rich element and MYB), defense response (TC-rich repeats), heat stress response (STRE), wounding and pathogen response (WRE3 and WUN-motif), metal response (O2-site and AP-1), anoxic response (ARE and G-C motif), low-temperature response (LTR), and drought response (MBS, DRE core, MYC, MYB recognition site, and as-1). The phytohormone response elements included those responding to ethylene (ERE and W-boxes), IAA (AuxRE-core, TGA-elements, and TGA-box), ABA (ABRE, ABRE3a, ABRE4, and CARE), gibberellins (TATC-box and P-box), MeJA response (TGACG-motif and CGTCA-motif), and salicylic acid (TCA-element). The light response elements included the ACE, GT1-motif, Sp1, MRE, ATC-motif, ATCT-motif, chs-CMA2a, AE-box, GA-motif, AT1-motif, TCT-motif, LAMP-element, TCCC-motif, I-box, GATA-motif, Gap-box, and Box 4. Other elements, such as the element (CAAAGATATC) were also identified, which is regarded as a cis-acting regulatory element involved in circadian control. Additionally, we predicted some tissue-specific preferentially expressed elements (RY-element, GCN4_motif, and HD-Zip 1). In general, the *CqACS* genes might be widely involved in mediating responses to hormone and stress response.

### 2.6. Analysis of CqACS Expression Patterns in Tissues

**Figure 6:**
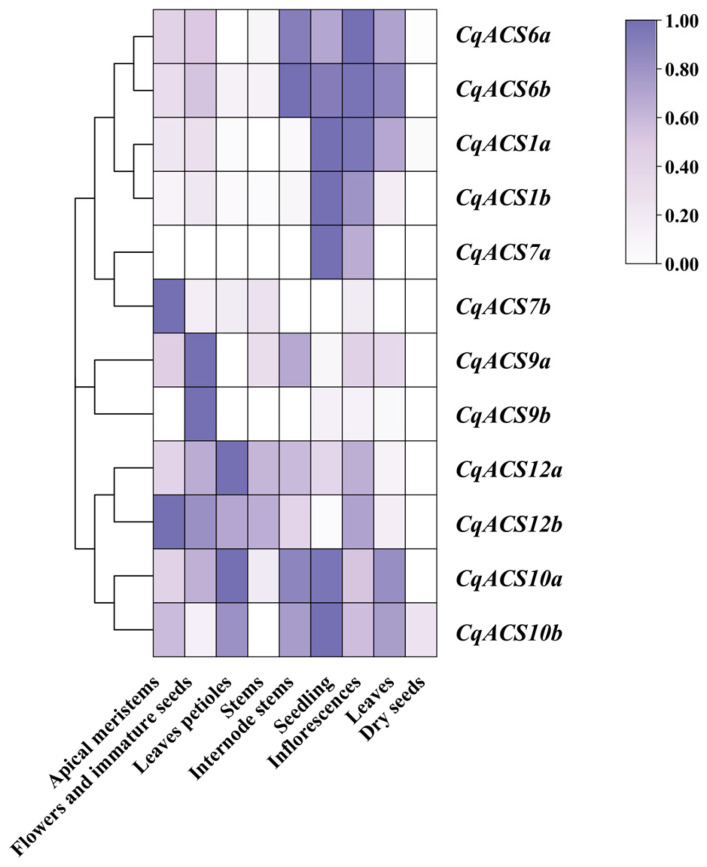
Expression patterns of *CqACS* genes in different tissues. The heatmap shows the expression levels of *CqACS* genes in nine tissues, including apical meristems, flowers and immature seeds, leaves petioles, stems, internode stems, seedling, inflorescences, leaves, and dry seeds.

To study the potential function of CqACS genes in C. quinoa, we retrieved and re-analyzed RNA-Seq data from the NCBI SRA database. As shown in Figure 6, all 12 CqACSs exhibited differential expression patterns across nine different tissues. CqACS2b, CqACS7a, and CqACS10b showed specific and high expression levels in inflorescences; CqACS9a and CqACS9b were specifically and highly expressed in flowers and immature seeds; CqACS12a and CqACS12b exhibited specific and high expression in leaves, petioles, and apical meristems, respectively. These results suggest distinct tissue-specific expression patterns among the CqACS genes.

### 2.7. Analysis of CqACS Genes Expression Patterns under Abiotic Stress

**Figure 7:**
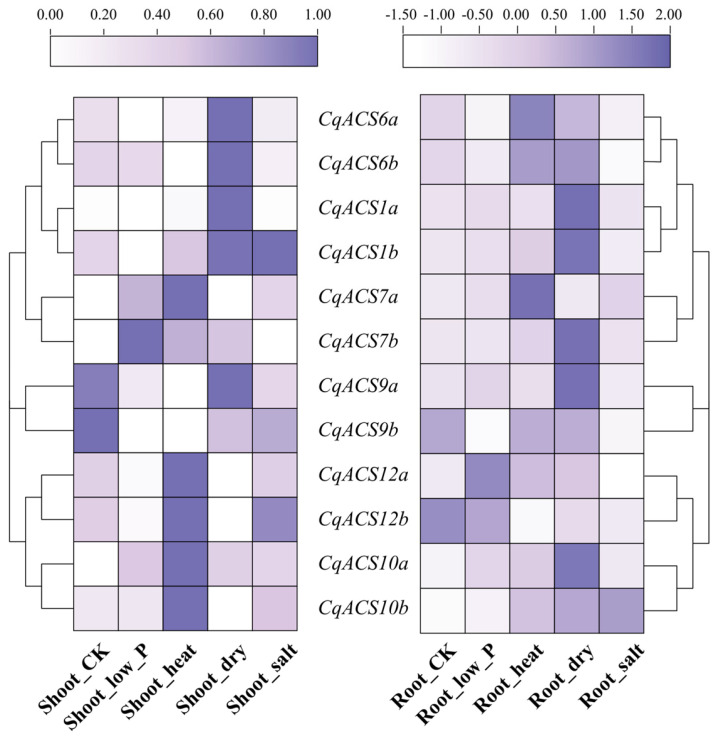
Heatmap of *CqACS* genes expression in shoot and root tissues of quinoa under abiotic stress.

Ethylene plays an important role in environmental stress, and the *ACS* gene encodes enzyme in the ethylene biosynthesis pathway [62,63,64]. In this study, we used transcriptome data to analyze the expression levels of *CqACS* genes in roots and shoots under abiotic stresses, including low phosphorus, high temperature, drought, and salt (Figure 7). We found that *CqACS7a*, *CqACS7b* in shoots and *CqACS12a*, *CqACS12b* in roots could respond to low phosphorus stress. Under heat stress, *CqACS7a*, *CqACS10a*, *CqACS10b*, and *CqACS12a* in shoots, and *CqACS6a*, *CqACS6b*, *CqACS7a* in roots exhibited significantly higher expression levels compared to the control. The expression of some genes in roots and shoots (*CqACS1a/1b*, *CqACS6a/6b*, *CqACS9a*) was significantly higher than that of control under drought stress. Under salt stress, the expression of *CqACS1b* and *CqACS12b* in shoots and *CqACS10b* in roots were significantly higher than the control. Overall, the expression of most *CqACSs* assessed were shown to respond to at least one abiotic stress treatment.

**Figure 8:**
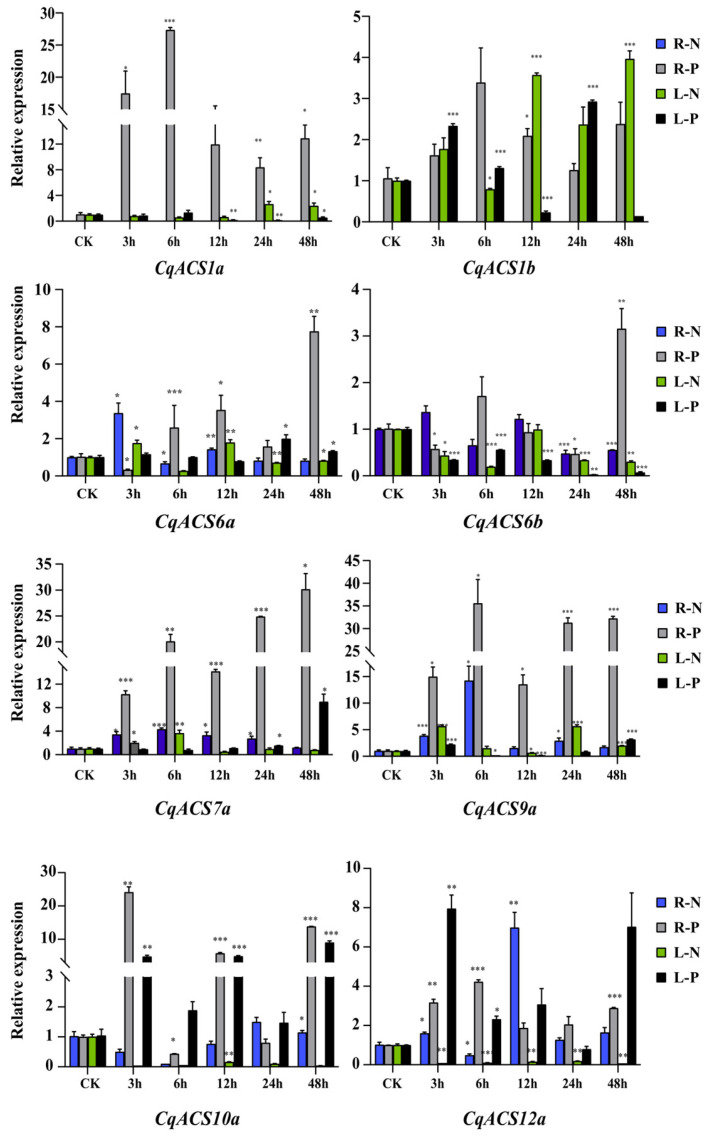
Expression analyses of *CqACS* genes under different abiotic stress conditions by qRT-PCR. Leaves and roots were collected from *C*. *quinoa* treated with 300 mM NaCl and 20% PEG 6000 stresses, respectively. R-N (root under the treatment of 300 mM NaCl); R-P (root under the treatment of 20% PEG 6000); L-N (leaves under the treatment of 300 mM NaCl); and L-P (leaves under the treatment of 20% PEG 6000). The *CqTub* gene was used as an internal control. The y-axis represents relative expression, calculated using the 2^−ΔΔCt^ formula. Student’s *t*-test: * *p* < 0.05; ** *p* < 0.01; and *** *p* < 0.001.

We also conducted qRT-PCR experiments to analyze the expression patterns of *CqACS* genes under drought (polyethylene glycol, PEG) and salt (NaCl 300 mM) stress in detail (Figure 8). The analysis of nucleotide sequences of *CqACSs* showed high similarities between *CqACS7a* and *CqACS7b*; *CqACS9a/b*, *CqACS10a/b* and *CqACS12a/b*. So *CqACS1a*, *CqACS1b*, *CqACS6a*, *CqACS6b*, *CqACS7a*, *CqACS9a*, *CqACS10a*, and *CqACS12a* were chosen for analysis. As the results show in Figure 8. Under drought stress conditions, *CqACS1a* and *CqACS1b* exhibited significant upregulation in the roots at all five treatment times, with the highest relative expression level observed in *CqACS1a* after 6 h of stress treatment. *CqACS6a* and *CqACS6b* also showed significant upregulation in the roots at 48 h of treatment. Both *CqACS7a* and *CqACS9a* showed significant upregulation in the roots at different stress treatment times, particularly *CqACS9a*, which reached a peak expression level after 6 h of stress treatment. *CqACS10a* and *CqACS12a* displayed significant upregulation in both roots and leaves at almost all different stress treatment times. Under salt stress conditions, the relative expression levels of *CqACS1a* were low, especially in the roots where *CqACS1a* and *CqACS1b* gene expressions were nearly undetectable. In contrast, *CqACS1b* showed significant upregulation in the leaves at all treatment time points, with the highest relative expression level observed after 48 h of stress treatment. However, *CqACS6a*, *CqACS6b*, *CqACS7a*, and *CqACS9a* exhibited upregulation in both roots and leaves at all treatment time points. Particularly, *CqACS9a* showed a significant increase in relative expression level in the roots after 6 h of stress treatment. *CqACS10a* and *CqACS12a* showed low relative expression levels in the leaves at all five treatment time points. However, there appeared to be some differences in the roots. *CqACS10a* exhibited upregulation in the roots after 24 and 48 h of stress treatment compared to the control. On the other hand, *CqACS12a* showed significant upregulation in the roots at almost all five treatment time points, particularly after 12 h of salt stress treatment. These results suggest that *CqACS* genes might play important roles in responding to drought and salt stresses in *C. quinoa*.

## 3. Discussion

ACS serves as a crucial enzyme within the internal ethylene biosynthesis pathway, acting as a rate-limiting factor that governs the synthesis of ethylene and the subsequent transmission of signals. This process helps regulate different stages of plant development and coordinate responses to stress. Until now, the clustering of *ACS* genes as a multigene family was recognized and thoroughly investigated in numerous plant species. The outcomes of genome screening unveiled the presence of 12 *ACS* genes in *A. thaliana* [23], 9 *ACS*-like genes in *H. brasiliensis* [65], 14 *ACS* genes in bananas [66], 12 *ACS* genes in wheat [67], as well as 18 *GaACS*, 35 *GhACS,* and 18 *GrACS* genes within *G. arboreum*, *G. hirsutum*, and *G. raimondii* [68]. In this investigation, a total of 12 *ACS* genes were detected within the genome of *C. quinoa*, distributed across 8 chromosomes, with 2 *ACS* genes on chromosomes 1, 7, 9, 13, and 16. Consistent with previous studies, the *ACS* genes exhibited an uneven distribution across multiple chromosomes. Analyzing their sequences and phylogenetic relationships revealed that among these *ACS* genes, eight isozymes (*CqACS1a*, *CqACS1b*, *CqACS2a*, *CqACS2b*, *CqACS7a*, *CqACS7b*, *CqACS9a,* and *CqACS9b*) demonstrated resemblance to the eight AtACS counterparts known for their ACS activity. The gene structure and distribution of motifs provided further corroboration for the results of the phylogenetic analysis. Based on the presence or absence of phosphorylation motifs located at the C-terminus, ACS proteins can be divided into three groups [23,59]. In our study, CqACS1a and CqACS1b belong to type 1 ACS isozymes and have three mitogen-activated protein kinases (MAPKs) phosphorylation sites and a single calcium-dependent protein kinases (CDPKs) phosphorylation site in the C-terminal region. A previous study showed that LeACS2 was phosphorylated at conserved serine residue Ser460 by CDPK under a wound-induced signal. This phosphorylation event led to heightened ACS activity and an elevated ACC content [69]. In *Arabidopsis*, the phosphorylation of type 1 ACS2 and ACS6 by MPK6 led to an increase in these ACS proteins, thereby increasing ACS activity and ethylene biosynthesis levels [70,71]. CqACS9a and CqACS9b belong to type 2 ACS isozymes. These two isozymes possess a single CDPK phosphorylation site and a distinct regulatory motif designated as “Target of ETO1” (TOE) situated in the C-terminal region. The TOE motif (WVF, RLSF, and R/D/E-rich amino acid motifs) mediates interaction with ETO1 E3 ligase and EOL1 and EOL2 (ETO1-like). These ETO1/EOL1/EOL2 E3 ligases containing BTB/TRP domains control the degradation of type 2 ACS proteins through 26S proteasomes [60,72]. Through biochemical and physiological studies, it was found that CK1.8 is involved in the phosphorylation of AtACS5 at position 463 of threonine. This phosphorylation event facilitates the association of AtACS5 with the E3 ubiquitin ligase ETO1, subsequently leading to the degradation of the AtACS5 protein [73]. Type 3 isozymes, CqACS7a and CqACS7b, lack the target sites required for protein phosphorylation in the C-terminal tail. However, a study demonstrated that AtCDPK16 could phosphorylate AtACS7, a type 3 ACS, at Ser216, Thr296, and Ser299 [18]. Ser212, Thr291, and Ser294 in CqACS7a and Ser226, Thr305, and Ser308 in CqACS7b correspond to Ser216, Thr296, and Ser299 in *Arabidopsis*, respectively, which are highly conserved. Further studies are needed to determine whether CqACS7a and CqACS7b are phosphorylated by CDPK. Efficient synthesis of ethylene as a stress signal helps plants and other sessile organisms adapt to harsh environments. The post-translational modification of ACS is an important regulatory mechanism to rapidly modulate ethylene levels in response to various stresses.

In most plant species, members of the ACS gene family exhibit distinct regulatory patterns at the transcriptional level, often in a manner that is specific to certain organs, tissues, or cell types. In Arabidopsis, the expression of all genes, excluding ACS9, is evident in young, etiolated seedlings or light-grown seedlings, while ACS9 expression is detected later. In mature Arabidopsis plants, ACS1 is mainly expressed in the flower stem, leaf vascular tissue, and central leaf veins; ACS2, 4, 5, 6, and 8 are expressed in younger leaves, siliques, inflorescence stem, and roots; ACS11 is expressed in younger leaves, cauline leaves, inflorescence stems, and in the roots, but ACS9 is barely expressed [74]. In our study, we found *CqACSs* were differentially expressed in the leaf petioles, apical meristems, flowers, immature seeds, seedlings, stems, internode stems, inflorescences, leaves, and mature seeds.

Many studies showed that *ACS* genes were responsive to various biotic and abiotic stresses, including fungal elicitor, wounding, salt stress, freezing, drought stress, and anaerobic conditions. Long term exposure to salt stress can significantly induce the expression of *GhACS1* in cotton [68]. In rice, the expression of *OS-ACS1* is induced by partial submergence, while the expression of *OS-ACS2* is inhibited by partial submergence [75]. Anaerobiosis can induce the expression of *OS-ACS1* in shoots and *OS-ACS3* in roots [76]. The *AtACS5* responds to salt stress, high temperature, and wounding, while the *AtACS7* responds to ABA and salt stress [31]. In wheat, *TaACS1*/*3*/*6*/*7*/*9*/*10* was induced by drought stress [67]. Notably, in this study, it was observed that the promoter regions of *CqACS* genes contained numerous elements associated with responses to both abiotic and biotic stresses, such as water stress, wounding and pathogen response, low temperature, heat stress, metal response, and anoxic response. These findings suggest that the *CqACS* genes might be involved in responses to various biotic and abiotic stresses. In our study, the expressions of most assessed *CqACSs* were shown to respond to at least one abiotic stress. *CqACS1a/1b/6a/6b* was significantly induced under drought stress, and their promoter contained many numerous elements associated with the response to drought stress, such as ATBP-1, MYB, MYC, MBS, or ABRE. *CqACS10a*, *CqACS10b*, *CqACS12a*, and *CqACS12b* clustered with *AtACS10* and *AtACS12*, which were considered to be amino acid transferases without ACS activity and the function of them was largely unknown. In cucumber, *CsACS10* and *CsACS12*, which grouped with *AtACS10* and *AtACS12*, were abundantly expressed in leaves, cotyledons, and tendrils [77]. Likewise, *CmaACS1* and *CmaACS9* grouped together with *AtACS10* and *AtACS12* exhibited higher expression levels in male flowers, and ethylene treatment inhibited the expression of *CmaACS9* in *C. maxima* [78]. In the present study, we found that *CqACS10a*, *CqACS10b*, *CqACS12a*, and *CqACS12b* were expressed in apical meristems, leaves petioles, and seedling, and their expressions were induced by abiotic stress. These findings suggest that the *CqACS10a*, *CqACS10b*, *CqACS12a*, and *CqACS12b* genes might be involved in plant development and response to abiotic stress. *CqACS10a* and *CqACS12a* were significantly up-regulated after exposure to PEG in roots and leaves, and *CqACS12a* was also significantly up-regulated in roots under NaCl treatment. The analysis of *CqACS* gene expression under abiotic stresses implies that the function of *CqACSs* was complex, providing clues for further research on the role of ethylene in abiotic stress tolerance of *C. quinoa.*

## 4. Materials and Methods

### 4.1. Plant Materials and Abiotic Stress Treatments

The quinoa material used in this experiment was YT077. The quinoa seeds were sown in pots containing sterilized mixed soil (1 part vermiculite: 3 parts soil) and were grown under controlled conditions at 22 °C with a photoperiod of 16 h light and 8 h dark for 20 days. After the initial growth period, well-grown samples were subjected to abiotic stress treatments. The abiotic stress treatments included drought (induced by 20% polyethylene glycol 6000, PEG) and salt stress (induced by 300 mmol/L NaCl) for different durations: 0, 3, 6, 12, 24, and 48 h, respectively. Untreated plants were used as control (CK). After the abiotic stress treatments, leaves and roots were collected. All collected samples were rapidly frozen in liquid nitrogen and stored at −80℃ until RNA extraction. The experimental design followed a randomized complete block design with three replications.

### 4.2. Prediction and Identification of CqACSs in C. quinoa

To identify potential *CqACSs* (1-aminocyclopropane-1-carboxylate synthase) in *Chenopodium quinoa*, protein sequences of known *Arabidopsis AtACS* family members were obtained from the Arabidopsis database TAIR (https://www.arabidopsis.org/index.jsp, accessed on 3 July 2022). The *C. quinoa* genome data were downloaded from Chenopodium DB (https://www.cbrc.kaust.edu.sa/chenopodiumdb/download/download-auth.html, accessed on 3 July 2022). Additionally, full-length protein sequences of known *ACS* genes from *Pyrus communis* (Pc), *Oryza sativa* (OS), and *Zea mays* (Zm) were retrieved from the GenBank Database (https://www.ncbi.nlm.nih.gov/genbank/, accessed on 3 July 2022).

Using the known AtACS amino acid sequences as query sequences, potential ACS protein sequences in *C. quinoa* were identified by performing searches with TBtools (V1.108) (https://github.com/CJ-Chen/TBtools/releases, accessed on 5 July 2022) using an E-value cutoff of ≤10^−5^. Furthermore, a hidden Markov model (HMM) of the “Aminotran_1_2” (PF00155) domain, retrieved from the Pfam protein family database was employed for the identification of potential ACS protein sequences using the BlastP method, again using an E-value cutoff of ≤10^−5^. The sequences obtained from both methods were intersected to identify the candidate CqACS proteins. To confirm the candidate proteins as CqACS family members, domain identification was performed using NCBI’s CDD tool (https://www.ncbi.nlm.nih.gov/cdd/, accessed on 5 July 2022). Only sequences containing the “Aminotran_1_2” (PF00155) domain were considered as final ACS proteins. Isoelectric points, molecular weights, and subcellular location predictions were acquired from the ExPasy website (http://web.expasy.org/protparam/, accessed on 5 July 2022), while subcellular locations were further predicted using the WOLF PSORT II online server (https://www.genscript.com/wolf-psort.html, accessed on 5 July 2022).

### 4.3. Phylogenetic Analysis

For investigating the phylogenetic relationships among *CqACS* genes, we utilized full-length ACS protein sequences from *C. quinoa* (Cq), *Pyrus communis* (Pc), *Oryza sativa* (OS), *Zea mays* (Zm), and *Arabidopsis thaliana* (At) in the phylogenetic analysis. The phylogenetic tree was created utilizing the neighbor-joining (NJ) method with the Poisson model, employing partial deletion. The process involved 2000 bootstrap replicates and was conducted using MEGA7.0 software (v7.0.26) provided by Mega Limited, Auckland, New Zealand (https://www.megasoftware.net/, accessed on 6 July 2022). The results are visualized and presented using iTOL V6 (https://itol.embl.de/, accessed on 6 July 2022).

### 4.4. Sequence Analysis and Structural Features

Full-length *C. quinoa* protein sequences underwent analysis through Multiple Expectation Maximization for Motif Elicitation (http://meme-suite.org, accessed on 6 July 2022) to discover conserved motifs. The maximum number of motifs was set to 15. Gene exon–intron structure characteristics were analyzed by aligning open reading frames (ORFs) with their genomic DNA sequences, and the TBtools (V1.108) (https://github.com/CJ-Chen/TBtools/releases, accessed on 6 July 2022) was used for visualization. The amino acid sequences encoded by the *CqACS* genes were aligned using MEGA 7.0 with the ClustalW2 program (V7.0.26) and presented with GENEDOC (V2.7) software.

### 4.5. Investigation of Collinearity and Selection Pressure in the ACS Gene Family

Gene duplication events were detected using the One Step MCScanX tool in TBtools. The gene density of the genomes, positions of *CqACSs* on the chromosomes, and gene duplication relationships were visualized using the Advanced Circos feature in TBtools. Additionally, the built-in McScanX software in TBtools was utilized to conduct collinearity analysis on the ACS genes of *A. thaliana* and *C. quinoa*, and the relationship diagram was generated using the TBtools toolkit. To assess selective pressure, the Ka/Ks Calculator in TBtools was utilized to calculate non-synonymous (ka) and synonymous (ks) substitutions.

### 4.6. Promoter Sequence Analysis

The promoter sequences of *CqACS* family members were identified by selecting the upstream 2000 bp regions from the start codons. For predictive analysis of plant cis-acting regulatory elements in these sequences, Plant CARE (http://bioinformatics.psb.ugent.be/webtools/plantcare/html/, accessed on 6 July 2022) was employed. Subsequently, TBtools was utilized to visualize the positions of the identified promoters.

### 4.7. Analysis of CqACS Gene Expression Patterns

The transcriptome data of different tissues and organs of quinoa (No: PRJNA394651) and different treatments (No: PRJNA306026) were obtained from the Bioproject database (http://www.ncbi.nlm.nih.gov/sra, accessed on 6 July 2022). RNA-seq data in TPM (transcripts per million reads) are normalized and performed log_2_ conversion. The visualization of the heatmap was accomplished using Tbtools.

### 4.8. Fluorescence Quantitative RT-qPCR Experiment and Data Evaluation 

RNA was extracted using the TransZol method, and the first cDNA was synthesized using HiScript II Q Select RT SuperMix for subsequent qRT-PCR analysis. All the reagents mentioned above were obtained from Novizan Biotechnology Co., Ltd. (Nanjing, China). Quantitative PCR was performed using QIAGEN (Hilden, Germany) Rotor-Gene Q to detect gene expression levels. The quinoa *Tubulin* (*CqTub*) gene was used as the internal standard gene. Primer sequences for *CqACS* family members and *CqTub* were listed in Table S5. We used Primer3 software to design primers in the non-conserved region of the *ACS* genes, resulting in amplicon sizes of around 200 bp. The specificity of primers was detected by agarose gel electrophoresis and melting curve analysis. In addition, PCR products were sequenced to determine the specificity of primer pairs. Each reaction was conducted in biological triplicates, and the data obtained from real-time PCR amplification were analyzed using the 2^−ΔΔCT^ method [79]. GraphPad Prism software was employed to create bar graphs representing the expression levels of shoots and roots under abiotic stress conditions, allowing for the analysis of the expression patterns of *CqACS* genes.

## 5. Conclusions

In this study, we identified 12 *CqACS* (1-aminocyclopropane-1-carboxylate synthase) genes from the quinoa genome, which clustered into four groups. Members within the same group exhibited similar structures and conserved motifs. We investigated the expression profiles of these genes in different tissues and under various abiotic stress conditions, and we found that these genes were responsive in different tissues and showed distinct responses to various types of abiotic stresses. Through this research, we aim to explore the potential roles of the *ACS* gene family in quinoa’s growth, development, and response to abiotic stress. It is our hope that these findings will provide valuable insights for the promotion and cultivation of quinoa in diverse environments.

## Figures and Tables

**Table 1:** The characteristics of 12 *ACSs* in *Chenopodium quinoa*.

Gene ID	Gene Name	Chromosome Position	Theoretical pI	Number of Amino Acid	Molecular Weight	Grand Average of Hydropathicity	Subcellular Localization
AUR62022615	CqACS1a	Chr10:4651267–4654352	7.23	497	55,985.93	−0.288	Nucleus
AUR62004409	CqACS1b	Chr01:118455577–118464122	6.71	512	57,651.79	−0.296	Nucleus
AUR62010746	CqACS6a	Chr13:8205683–8205683	5.94	483	53,848.55	−0.128	Chloroplast
AUR62019750	CqACS6b	Chr16:70595450–8208172	5.77	483	53,939.62	−0.145	Chloroplast
AUR62031241	CqACS7a	Chr01:22940624–22942199	5.27	445	50,266.87	−0.291	Cytosol
AUR62027462	CqACS7b	Chr02:42232745–42240825	5.73	459	51,655.74	−0.225	Vacular membrane
AUR62004042	CqACS9a	Chr09:9375889–9377517	7.99	468	52,418.95	−0.251	Nucleus
AUR62007557	CqACS9b	Chr09:487147–488780	7.99	468	52,432.98	−0.25	Nucleus
AUR62010540	CqACS10a	Chr13:11554392–11557248	7.08	548	60,330.16	−0.092	Plasma membrane
AUR62017355	CqACS10b	Chr16:66543418–66545916	7.5	547	60,162.96	−0.123	Plasma membrane
AUR62035045	CqACS12a	Chr11:72840752–72843517	6.11	359	39,788.84	0.018	Cytoskeleton
AUR62016097	CqACS12b	Chr07:76925605–76928826	6.04	440	48,245.24	0.012	Chloroplast

## Data Availability

The data presented in this study are available in the article and Supplementary Materials.

## Supplementary Materials

The following supporting information can be downloaded at: https://www.mdpi.com/article/10.3390/plants12234021/s1, Figure S1: Amino acid sequence alignment of CqACS and AtACS proteins. The rectangles indicate the seven highly conserved regions (Boxes 1–7). The conserved glutamate residue (E) marked with a filled circle is involved in substrate specificity. The open circles indicate the 11 amino acids conserved among ACS isozymes and various amino transferases. RLSF motifs were marked with rectangles (CDPK), WVF motifs were marked with rectangles in blue. The Serine residues that in “RLSF” motif and long C-terminal were marked with red color; Table S1: Analysis of the 15 conserved motifs of CqACS proteins in *C. quinoa;* Table S2: Segmentally duplicated *CqACS* gene pairs; Table S3: One-to-one orthologous relationships between *C. quinoa* and *A. thaliana;* Table S4: Information of cis-element in *CqACSs* promoter region; Table S5: qRT-PCR Primer.
