# Supplementary material for: Genome-Wide Identification and Expression Analysis of 1-Aminocyclopropane-1-Carboxylate Synthase (ACS) Gene Family in Chenopodium quinoa
Source: Plants (Basel). 2023 Nov 29;12(23):4021. doi: 10.3390/plants12234021 (PMC10707884; doi:10.3390/plants12234021)
Supplement: Supplementary file 1 [file plants-12-04021-s001.zip › Captions for SUPPLEMENTARY MATERIAL.pdf]

Figure S1. Amino acid sequence alignment of CqACS and AtACS proteins. The rectangles indicate the seven highly conserved regions (Boxes 1-7). The conserved glutamate residue (E) marked with a filled circle is involved in substrate specificity. The open circles indicate the 11 amino acids conserved among ACS isozymes and various amino transferases. RLSF motifs were marked with rectangles (CDPK), WVF motifs were marked with rectangles in blue. The Serine residues that in "RLSF" motif and long C-terminal were marked with red color.

Table S1 Analysis of the 15 conserved motifs of CqACS proteins in *C.quinoa*

Table S2 Segmentally duplicated *CqACS* gene pairs

Table S3 One-to-one orthologous relationships between *C.quinoa* and *A.thaliana*

Table S4 Information of cis-element in *CqACSs* promoter region

Table S5 qRT-PCR Primer
