# Supplementary material for: Genome-Wide Identification and Expression Analysis of 1-Aminocyclopropane-1-Carboxylate Synthase (ACS) Gene Family in Chenopodium quinoa
Source: Plants (Basel). 2023 Nov 29;12(23):4021. doi: 10.3390/plants12234021 (PMC10707884; doi:10.3390/plants12234021)
Supplement: Supplementary file 1 [file plants-12-04021-s001.zip › Figure S1 Amino acid sequence alignment of CqACS and AtACS proteins.pdf]

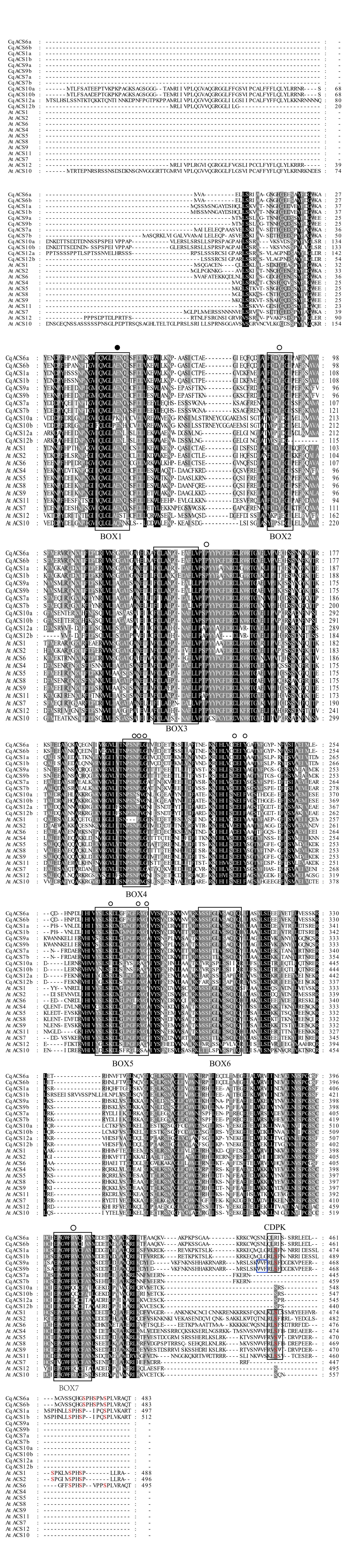
BOX1
BOX2
BOX3
BOX4
BOX5
BOX6
CDPK
BOX7
